# Effects of Tibetan medicine metacinnabar (β-HgS) combined with imipramine or sertraline on depression-like symptoms in mice

**DOI:** 10.3389/fphar.2022.971243

**Published:** 2022-09-02

**Authors:** Yajun Qiao, Cen Li, Ming Zhang, Xingfang Zhang, Lixin Wei, Keshen Cao, Xiaoyuan Zhang, Hongtao Bi, Tingting Gao

**Affiliations:** ^1^ Department of Psychiatry, The People’s Hospital of Jiangmen, Southern Medical University, Jiangmen, China; ^2^ Qinghai Provincial Key Laboratory of Tibetan Medicine Pharmacology and Safety Evaluation, Northwest Institute of Plateau Biology, Chinese Academy of Science, Xining, China; ^3^ Department of Psychology, School of Public Health, Southern Medical University, Guangzhou, China; ^4^ CAS Key Laboratory of Tibetan Medicine Research, Northwest Institute of Plateau Biology, Chinese Academy of Sciences, Xining, China; ^5^ Medical College, Qinghai University, Xining, China

**Keywords:** depression, β-HgS, imipramine, sertraline, combination therapy

## Abstract

Depression is a common mood disorder that has exhibited an increased incidence rate worldwide, but the overall clinical efficacy of antidepressants remains unsatisfactory. In traditional Ayurveda and Tibetan medicines, β-HgS-containing medicines have been used to treat neurological diseases for thousands of years, and our previous study found that β-HgS ameliorated depression-like behaviors in chronic restraint stress (CRS)-treated or chronic unpredictable mild stress (CUMS)-treated mice. Hence, present study investigated the effects of β-HgS combined with the clinical first-line antidepressants, imipramine (IMI) and sertraline (SER), on depression-like symptoms in CRS- and CUMS-co-treated mice. Our results revealed that β-HgS promoted the antidepressant effect of SER on depression-like behavior in mice, and enhanced its effects on promoting glucocorticoid receptor (GR) expression and neuronal proliferation in key hippocampal subregions, as well as increasing interleukin 10 (IL-10) levels and decreasing malondialdehyde levels in the sera of stress-stimulated mice. As for IMI, β-HgS enhanced its effects on preventing atrophy and severe structural damage in the hippocampus, as well as in promoting hippocampal GR levels and neuronal proliferation and serum IL-10 and superoxide dismutase (SOD) levels. Additionally, combination therapy resulted in the increased diversity of important intestinal microbiota compared to that of monotherapy, which may help sustain the health of the digestive tract and reduce inflammation to further enhance the antidepressant effects of IMI and SER in mice.

## 1 Introduction

The most intuitive manifestation of depression is a persistent sense of sadness and loss of interest. This emotional disorder can even affect the way an individual feels, thinks, and behaves, and can lead to various psychological and physical problems (Ferrari et al., 2013). With lifestyle and psychosocial environmental changes in modern society, the incidence of depression has continued to increase worldwide. At present, about 350 million people in the world suffer from depression of varying degrees, and depression is expected to become the world’s major disease burden by 2030 (data from the World Health Organization 2012) (WHO, 2012; Gigantesco et al., 2019; Hamel et al., 2019).

Antidepressants are the initial treatment choice for patients with mild-to-moderate depression and the definitive choice for patients with major depression. At present, the clinical use of antidepressants consists of four classes of drugs: tricyclic antidepressants (TCAs), serotonin reuptake inhibitors (SSRIs), selective serotonin noradrenaline reuptake inhibitors (SNRIs), and monoamine oxidase inhibitors (MAOIs) (Cosgrove et al., 2012). Additionally, drugs with other mechanisms of action—including bupropion, nefazodone, ketones of letrozole, and rice nitrogen equality are also used. Among clinical first-line antidepressants, imipramine (IMI) and sertraline (SER) are two of the most commonly used antidepressants in China. IMI, a milestone in the development of antidepressants, represents the first clinically available TCA and has been widely used to treat endogenous depression and functional or reactive depression since it was first successfully synthesized in 1957. SER is commonly used to treat major depressive disorder (MDD), obsessive-compulsive disorder (OCD), panic disorder, post-traumatic stress disorder (PTSD), premenstrual dysphoric disorder (PMDD), and social anxiety disorder. The SER belongs to SSRI, which is a common psychotropic drug (López-Muñoz and Álamo, 2009). Although some studies have suggested that some mechanisms of action of antidepressants are superior to those of others, there have been no repeatable findings to confirm the clinical significance of these claims (Bayes and Parker, 2019). For most patients, antidepressant efficacy is similar between different types of drugs and among different drugs of the same class. Despite these findings, studies of sequential treatment of depression suggest that some residual symptoms, such as sadness, impaired functioning, and negative self-perceptions, persist in patients with complete remission after treatment (Steven and Maurizio, 2011). Therefore, these residual symptoms can make patients in complete remission more likely to relapse into depression (Nil et al., 2016; Bressington et al., 2019). Therefore, the identification and development of adjuvant therapies for existing antidepressants have become a major focus of research on the treatment of depression.

In traditional Ayurveda and Tibetan medicines, the cubic form of mercury sulfide (metacinnabar, also known as β-HgS) and β-HgS-containing medicines have been used to treat various chronic ailments for thousands of years, especially neurological diseases such as neuroinflammation, brain trauma, and stroke (Liu et al., 2018). Recently, we revealed that the Tibetan medicine, Zuotai (containing 54.5% β-HgS), and β-HgS significantly relieve depression-like behaviors in CRS-treated or CUMS-treated mice (Niu et al., 2016; Zhao et al., 2016; Zhao J. et al., 2018). However, the antidepressant effects of β-HgS as an adjuvant therapy for existing antidepressants remain unknown. Therefore, in this study, to investigate the effect of β-HgS combined with clinical first-line antidepressants on depression-like symptoms, we chose two types of antidepressants, IMI and SER, for use in combined pharmacotherapies to evaluate the antidepressant-like ability of β-HgS as an adjuvant therapy in CRS- and CUMS-treated mice. Additionally, through behavioral testing, we analyzed the effect of these different treatment modalities on depression-like symptoms in mice, and by analyzing some physiological indicators of depression (including neurotransmitters, brain-derived neurotrophic factors, inflammatory factors, and antioxidants), receptors in various subregions of the hippocampus, and gut microbiota to explore the corresponding underlying mechanisms.

## 2 Materials and methods

### 2.1 Animals

The experimental animals were 8-week-old male Kunming (KM) mice obtained from Gansu University of Traditional Chinese Medicine, China [Animal Production License No. SCXK (Gan) 2015-0005). Mice were placed in standard cages with wood shavings. The rearing environment was a closed an imal room with an ambient temperature of 22 ± 1°C, artificial lighting from 7:00 am to 19:00 pm, and mice were fed standard laboratory chow and distilled water ad libitum. Animal experiments in this study were in accordance with ARRIVE guidelines and were approved by the Animal Experimentation Committee of the Institute of Plateau Biology, Chinese Academy of Sciences (Lot: NWIPB20171106-01), and strict accordance with the National Institutes of Health Guide for the Care and Use of Laboratory Animals (NIH Publication No. 8023) , revised in 1978.

### 2.2 Drugs and reagents

β-HgS was purchased from Alfa Aesar Chemical Co., Ltd. (Haverhill, MA, United States) with a purity of 98%. IMI hydrochloride was obtained from the National Institutes for Food and Drug Control (Bejing, China). SER hydrochloride was purchased from Shanghai Yuanye Bio-Technology Co., Ltd. (Shanghai, China).

Enzyme-linked immunosorbent assay (ELISA) kits for 5-hydroxytryptamine(5-HT), norepinephrine (NE), and brain-derived neurotrophic factor (BDNF) for neurotransmitter detection; corticosterone (CORH), corticotropin-releasing hormone (CRH), and adrenocorticotropic hormone (ACTH) ELISA kits for the detection of HPA axis core indicators; interleukin-6 (IL-6), IL-10 and tumor necrosis factor-α (TNF-α) ELISA kits to detect cellular immune factors, the above ELISA kits were purchased from Jianglai Biotechnology Co., Ltd. (Shanghai, China); purchased from Nanjing Jiancheng Bioengineering Institute (Nanjing, China) detection kit for the detection of SOD, catalase (CAT) and malondialdehyde (MDA). Antibodies used in fluorescence immunohistochemistry were purchased from Abcam, Inc. (Cambridge, Cambridgeshire, United Kingdom), including anti-GR antibody (ab3578), anti-Proliferating Cell Nuclear Antigen (PCNA) antibody (ab18197), anti-Neuronal Nuclei (NeuN) antibody (ab128886), anti-NG2 antibody (ab129051) and anti-Olig2 antibody (ab136253). The chemicals in other laboratory tests are of analytical grade and manufactured in China.

### 2.3 Chronic predictable and unpredictable stress procedures and drug treatments

**FIGURE 1:**
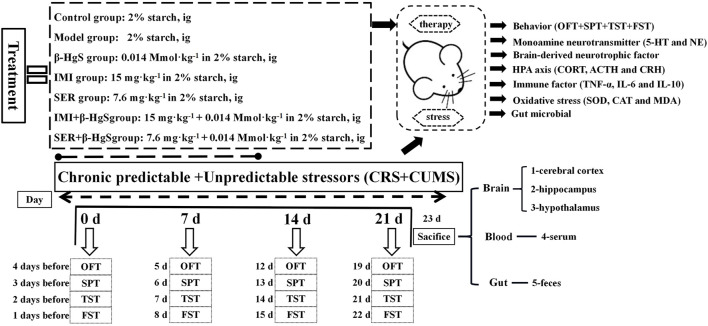
Experimental flowchart.

After 1 week of acclimatization, mice were randomly divided into the following seven groups (*n* = 10): 1) normal, 2) model, 3) β-HgS, 4) IMI, 5) SER, 6) IMI+β-HgS, and 7) SER+β-HgS. Mice in the normal and model groups were intragastrically administrated 2% (w/v) starch solution. β-HgS, IMI, and SER were separately dissolved in 2% (w/v) starch solutions and were then intragastrically administrated to mice in the drug-treatment groups (β-HgS, IMI, SER, IMI+β-HgS, and SER+β-HgS groups) at dosages of 0.014 mmol.kg^−1^ (β-HgS) (Zhao J. et al., 2018), 15 mg.kg^−1^ (IMI) (Furukawa et al., 2022), and 7.6 mg.kg^−1^ (SER) (Weilburg et al., 2003) per day for 3 weeks. Drugs were prepared the day before the experiment and stored in a 4°C refrigerator. Additionally, the mice exposed to chronic predictable and unpredictable stressors included a predictable chronic restraint stressor and a total of seven unpredictable mild stressors per day for 3 weeks, with the exception of the normal group. The predictable chronic restraint stressor consisted of placing the mice into tubes from 8:30 to 14:30 each day (Ding et al., 2016). The experiment used seven different stimuli to simulate an unpredictable stressor [1): 5 min of heat stress at 42°C; 2): 2 min of cold stress at 10°C; 3): 2 min of reciprocating swing; 4): 24 h of 45° tilt; 5): 24 h of fasting; 6): 24 h of water deprivation; 7): 24 h of day-night reversal], and each stimulus occurred randomly, with three stimulations per day (Ma et al., 2018). In addition, the body weight of each group of mice was measured weekly (Figure 1).

### 2.4 Behavioral tests

The behavioral tests—including the sucrose preference test (SPT), open field test (OFT), tail suspension test (TST), and forced swimming test (FST)—were conducted before the stress procedures and drug treatments began (0 days) and at the end of the 3-week stress period (21 days). The SPT was performed out following the procedures described by Yan et al. (2015), and the sucrose preference ratio (SPR) was calculated as follows: SPR (%) = sucrose intake/total fluid intake (including sucrose and water intake); 2 h-food consumption = the amount of food before the test minus the amount of food after the test. The OFT was performed following the procedures described by Choleris et al. (2001). TST was performed following the procedures described by O'Leary and Cryan (2009). Finally, the FST was carried out following the procedures described by Porsolt et al. (1977), Qiao et al. (2020).

### 2.5 Determination of biomarkers related to depression, immune function, and oxidative stress

After the behavioral test, mouse blood was collected through the retro-orbital venous plexus, and the collected mouse blood was placed in coagulation tubes, respectively, and serum was obtained by centrifugation at 4°C and 4,000 RPM for 10 min. Immediately after blood collection, the animals were sacrificed, and the cortex, hippocampus, and hypothalamus were collected from the brain and mixed with phosphate-buffered saline (pH 7.2) in proportion to each, and these tissues were homogenized using a tissue homogenizer (frequency: 60 Hz), rotation rate: 1,800 times/min, duration: 2 min). The supernatant was then extracted after centrifugation for 10 min at 4°C and 5,000 RPM. According to the kit manufacturer’s instructions, 5-HT and NE content in the cortical supernatant, BDNF content in the hippocampal supernatant, CRH level in hypothalamic supernatant, as well as CORT, ACTH, TNF-α, IL-6, IL-10, MDA, CAT, and SOD levels in serum were determined, respectively.

### 2.6 Immunohistochemistry

Whole brains were collected from the control and stress-stimulation groups after the stress stimulations were completed. Briefly, the brains were fixed in 4% paraformaldehyde solution, paraffin-embedded, and serially sectioned at a slice thickness of 5 μm. Then, the paraffin-embedded sections were deparaffinized, and non-specific binding was blocked by incubation with 3% BSA for 30 min. Subsequently, brain sections were incubated with primary antibodies against PCNA (1:500), GR (1:500), NG2 (1:500), NeuN (1:200), and Olig-2 (1:250) overnight at 4°C. After incubation and washing, FITC-conjugated and Cy3-conjugated secondary antibodies were added. Following incubation with these antibodies (4°C, 24 h for primary antibodies; and room temperature, 45 min for secondary antibodies), the sections were embedded in Fluoroshield mounting medium with DAPI (ab104139, Abcam). Images were acquired using a fluorescence microscope (IX73, Olympus, Japan) (Qiao et al., 2020).

### 2.7 Intestinal microbial-diversity analysis

After the stress stimulations, fresh rectal contents of the mice were collected, frozen quickly in liquid nitrogen, and placed in a refrigerator at −80°C for subsequent DNA extraction. Microbial DNA was extracted using the E.Z.N.A. Stool DNA Kit (Omega Bio-tek, Norcross, GA, United States) according to the manufacturer’s protocols, after which the extracted DNA was stored at −80°C until further use.

The primers, 338F (5′-CTC​CTA​CGG​GAG​GCA​GCA​G-3′) and 806R (5′-GGACTACHVGGGTWTCTAAT-3′), were used to amplify the V3–V4 region of the bacterial 16S rDNA gene. The amplification procedure of PCR reactions was 95°C for 3 min, followed by 27 cycles at 95°C for 30 s, 55°C for 30 s, and 72°C for 45 s, as well as a final extension at 72°C for 10 min. Approximately 420 bp of amplified fragments was obtained per sample. PCR reactions were performed in 20 μl volumes containing 4 μl of 5 × FastPfu Buffer, 2 μl of 2.5 mM dNTPs, 0.8 μl of 5 μM primers, 0.4 μl of FastPfu Polymerase, and 10 ng of template DNA *via* a PCR system (ABI GeneAmp 9700, Applied Biosystems, Inc., MA, United States). Using the MiSeq platform, paired-end data of 2 × 300 bp were obtained *via* sequencing. Long sequences were obtained by splicing, and 16S analysis was performed. The raw reads were deposited into the NCBI Sequence Read Archive (SRA) database (https://www.ncbi.nlm.nih.gov/sra/?term=PRJNA850836).

The original sequence was quality-filtered using Trimmomatic software (version 0.39) and was spliced by FLASH software with the following criteria: 1) setting a 50 bp sliding window, the 300 bp reads were truncated at any site receiving an average quality score < 20 over this window, discarding the truncated reads that were shorter than 50 bp; 2) barcodes matching exactly 2-nucleotide mismatches in primer matching were allowed, and reads containing ambiguous characters were removed; and 3) two end sequences were spliced according to base overlap, whereas overlaps longer than 10 bp and unspliced sequences were removed. Reads that could not be assembled were discarded. Operational taxonomic units (OTUs) were clustered with a 97% similarity cutoff using UPARSE (version 7.1 http://drive5.com/uparse/), and chimeric sequences were identified and removed using UCHIME. The taxonomy of each 16S rRNA gene sequence was analyzed by RDP Classifier (http://rdp.cme.msu.edu/) against the Silva (SSU123)16S rRNA database using a confidence threshold of 70% (Amato et al., 2013; Qiao et al., 2020).

### 2.8 Statistical analysis

Experimental data for all tests are presented as the mean ± standard deviation (SD) of six replicates and an analysis of variance was performed for a completely randomized experimental design. Data were subjected to multiple t-tests using SPSS 22.0 software to identify differences between means (without assuming consistent SD) and “Pearson” correlation analysis. *p*-Value < 0.05 was considered statistically significant.

## 3 Results

### 3.1 Effect on body weight

**FIGURE 2:**
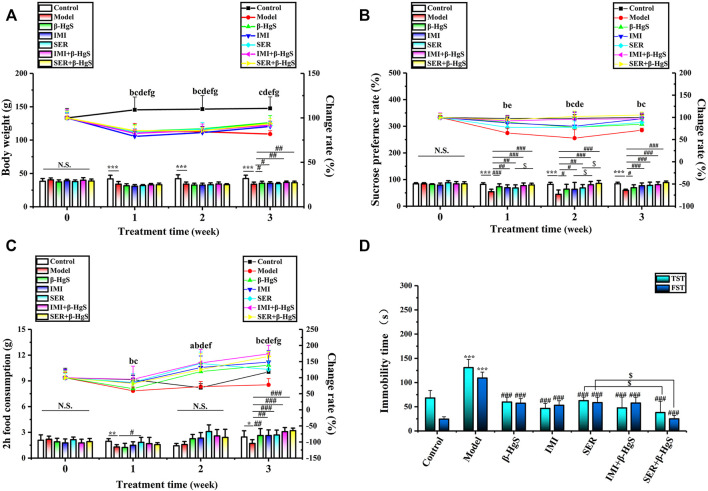
The effects of drug treatments on **(A)** body weight, **(B)** sucrose preference ratio, **(C)** 2 h-food consumption, and **(D)** immobility time in the tail suspension test (TST) and forced swimming test (FST) of stress-stimulated mice. Data are shown as the mean ± *SD* (*n* = 10). **p < 0.01 and ***p < 0.001 denote a significant difference from the control group. ^#^ *p* < 0.05, ^##^ *p* < 0.01, and ^###^ *p* < 0.001 denote significant differences from the model group. ^$^ *p* < 0.05 denotes a significant difference from the relative monotherapy group. Intra-group comparison of each group, the letters indicate significant differences compared with 0 days *p* < 0.05.

As shown in Figure 2A, mice in the model group significantly lost body weight due to experiencing stress stimuli, as their body weight losses were 19.52%, 20.85%, and 23.82% on days 7, 14, and 21, respectively, compared to those of the control group. In contrast, the body weights of the mice in the drug-treated groups all initially decreased, but then increased during the 3-week treatment and yielded significantly higher body weights compared those of the model group on day 21 (*p* < 0.05 or *p* < 0.01), except for the SER group. The mice in the IMI+β-HgS group had the highest body weights among all drug-treated groups on day 21.

### 3.2 Effects on sucrose preference ratio and two h-food consumption

SPR and food consumption are usually used to evaluate anhedonia levels (a core symptom of depression) in mice, and corresponding results in this study are shown in Figures 2B,C. Compared to those of the normal group, the SPR and 2 h-food consumption of mice in the model group were both dramatically decreased by 3-week stress stimulation, as these decreases were 28.96% and 30.46%, respectively. Compared with those of the model group, the SPR and 2 h-food consumption of mice in all drug-treated groups were increased significantly on day 21 (*p* < 0.05, *p* < 0.01, or *p* < 0.001). Moreover, the SPR of mice in the IMI+β-HgS group was significantly higher than that of the IMI group on day 14 (*p* < 0.05), and the SPR of mice in the SER+β-HgS group was significantly higher than that of the SER group on days 7 and 14 (*p* < 0.05).

### 3.3 Effects on immobility time in the TST and FST

The TST and FST are two of the primary methods for evaluating depression-like behavior in mice, in which the corresponding immobility time reflects the level of depression-like behavior. As shown in Figure 2D, 3-week stress stimulations significantly increased the immobility time of mice in the TST and FST in the model group (*p* < 0.001). Compared with that of the model group, the immobility time of mice in all drug-treated groups was significantly shorter in both the TST and FST (*p* < 0.001). In particular, the mice in the SER+β-HgS group had much shorter immobility times than those in the SER group in both the TST and FST (*p* < 0.001).

### 3.4 Effects on rest time, movement time, center residence time, and movement distance in the open field test

**FIGURE 3:**
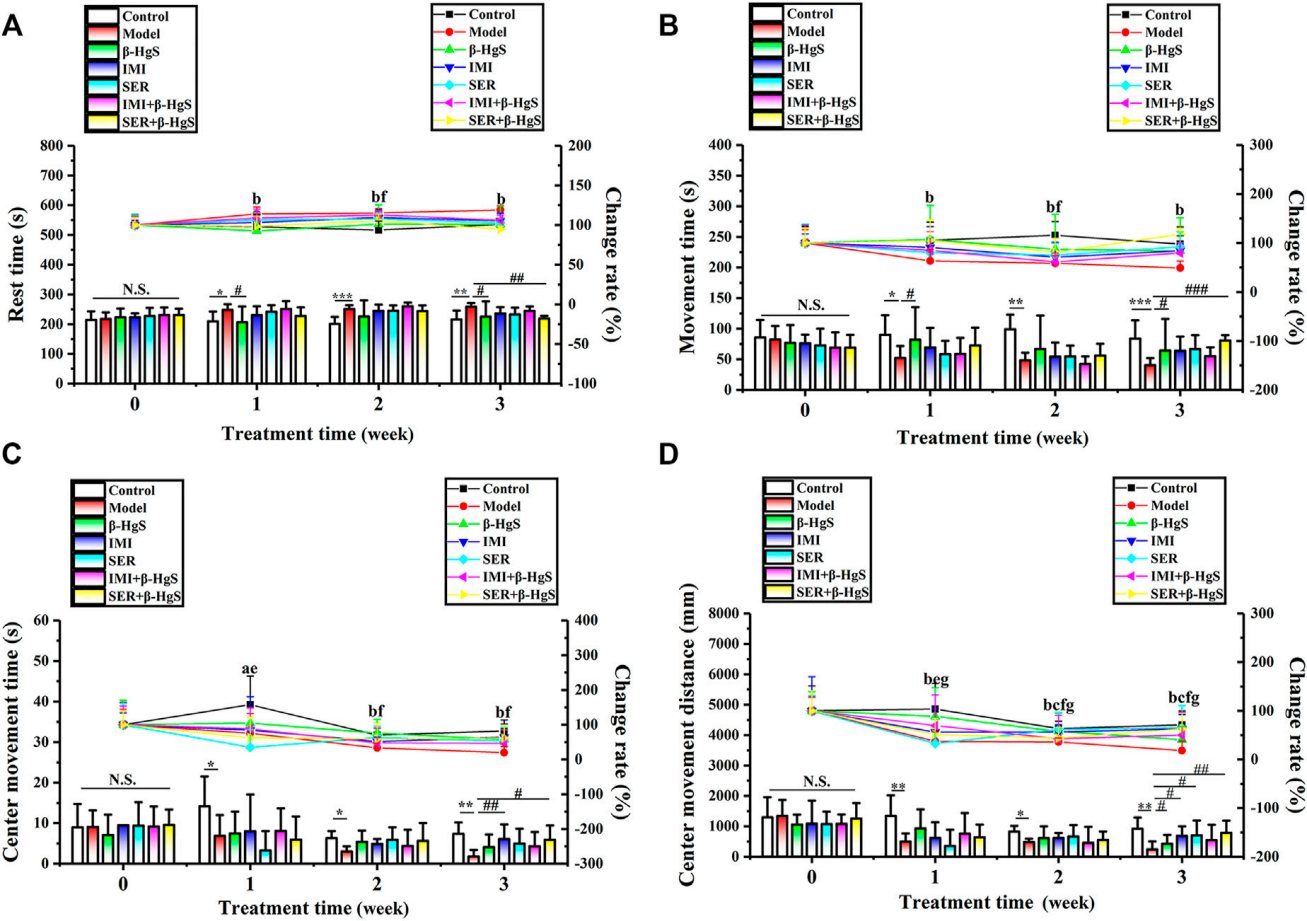
Effects of drug treatments on **(A)** rest time, **(B)** movement time, **(C)** center movement time, and **(D)** center movement distance in stress-stimulated mice in the open-field test. Data are shown as the mean ± *SD* (*n* = 10). *p < 0.05, **p < 0.01, and ***p < 0.001 denote a significant difference from the control group. ^#^ *p* < 0.05, ^##^ *p* < 0.01, and ^###^ *p* < 0.001 denote significant differences from the model group. Intra-group comparison of each group, the letters indicate significant differences compared with 0 days *p* < 0.05.

The activities of mice were tested using the OFT during the 3-week stress stimuli and drug treatments. As shown in Figure 3, compared to the parameters in the control group, the mice in the model group exhibited a marked increase in the rest time (RT) and dramatic decreases in movement time (MT), center residence time (CRT), and movement distance (CMD) on day 7 onward. Compared with parameters in the model group, mice had less RT and more MT and CMT in the β-HgS and SER+β-HgS groups (*p* < 0.05, *p* < 0.01, or *p* < 0.001), as well as more CMD in the β-HgS, IMI, SER, and SER+β-HgS groups (*p* < 0.05 or *p* < 0.01) on day 21. These results suggest that mice in the SER+β-HgS and β-HgS groups were the most active among all drug-treated groups.

### 3.5 Effects on morphological structure, GR levels, oligodendrocyte numbers, and neuronal proliferation in key hippocampal subregions

**FIGURE 4:**
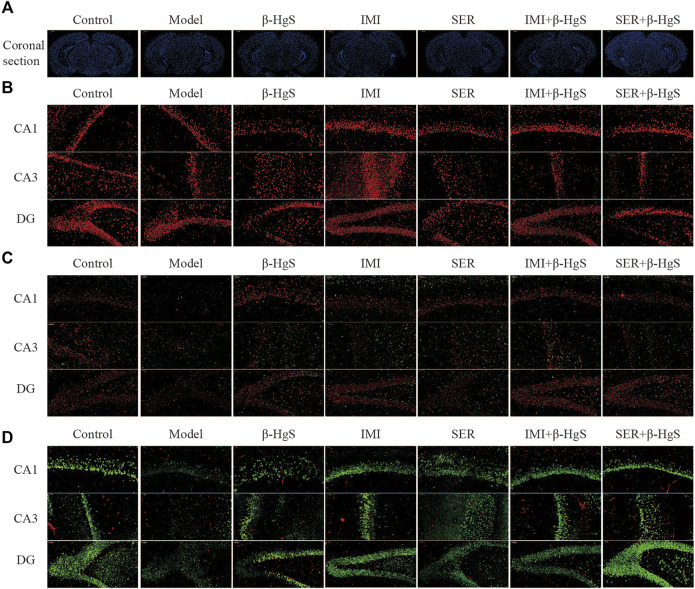
Effects of drug treatments on the structure, expression of GRs and oligodendrocytes, and neuronal proliferation in key hippocampal subregions of stress-stimulated mice. **(A)** Coronal sections, **(B)** immunofluorescent images of GRs (red), **(C)** immunofluorescent images of NG+ cells (red) and Olig2+ glial cells (green), and **(D)** immunofluorescent images of NeuN+ cells (green) and PCNA+ cells (red).

**FIGURE 5:**
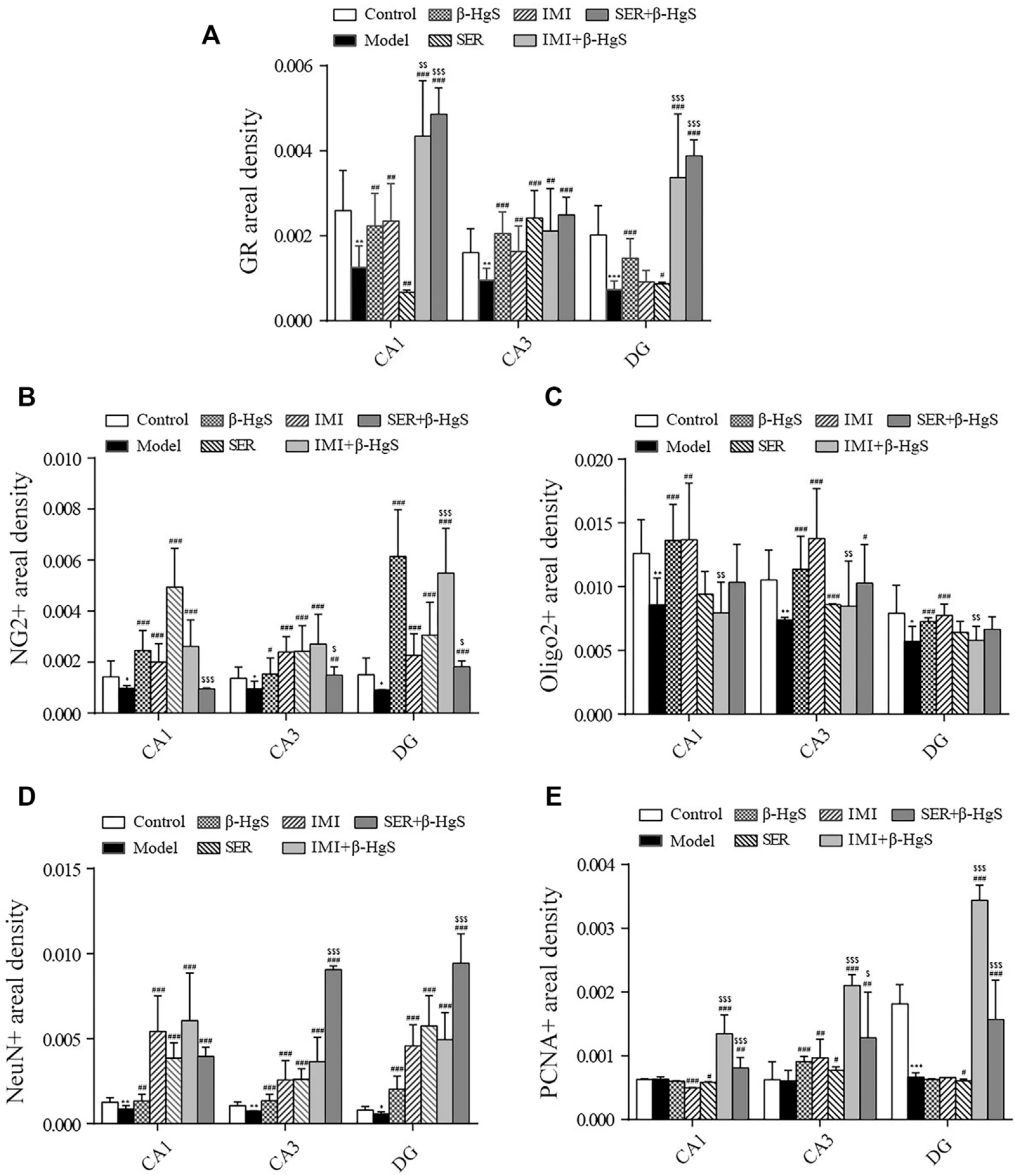
Effects of drug treatments on fluorescence intensities of GRs and oligodendrocytes, and neuronal proliferation in key hippocampal subregions of stress-stimulated mice. **(A)** Fluorescence intensity of GRs, **(B)** fluorescence intensity of NG2+ glial cells, **(C)** fluorescence intensity of Olig2+ glial cells, **(D)** fluorescence intensity of NeuN+ cells, and **(E)** fluorescence intensity of PCNA+ cells in subregions of the hippocampus. Data are shown as the mean ± *SD* (*n* = 4). *p < 0.05, **p < 0.01, and ***p < 0.001 denote significant differences from the control group. ^#^ *p* < 0.05, ^##^ *p* < 0.01, and ^###^ *p* < 0.001 denote significant differences from the model group. ^$^ *p* < 0.05, ^$$^ *p* < 0.01, and ^$$$^ *p* < 0.001 denote significant differences from the relative monotherapy group.

The effects of stress and drug treatments on morphological structure, GR levels, oligodendrocyte numbers, and neuronal proliferation in key hippocampal subregions were assessed via immunohistochemistry (Figures 4, 5). As shown in Figure 4A, the representative coronal section of the mouse brain shows the complete morphology and clear structure, and the hippocampal cells are arranged orderly in each subregion of the hippocampus of mice in the control group. Compared with those of the control group, the hippocampus of the mice in the model was atrophied and the hippocampal cells were disorganized and fewer in number, suggesting severe structural damage in the hippocampus of the mice in the model group. Compared with the characteristics in the model group, there was much less structural damage in the hippocampi of the mice in all drug-treatment groups, among which the structural damage of the hippocampus in the IMI+β-HgS group was less than that found in the other drug-treatment groups.

Immunofluorescent staining of GRs in key hippocampal subregions is shown in Figure 4B, and the corresponding fluorescence densities are shown in Figure 5A. Compared with features of the control group, the distribution of GR-positive cells was narrower, the cellular morphology was irregular and disorderly, and the fluorescence intensities were significantly weakened (*p* < 0.01 or *p* < 0.001) in all key subregions of the hippocampus in the model groups. Compared with properties of the model group, GR-positive cells exhibited a larger distribution range and significantly higher fluorescence intensities in key subregions of the hippocampus in drug-treatment groups, with the exceptions of CA1 in the SER group and the dentate gyrus (DG) in the IMI group. The fluorescence intensity of GR-positive cells in the combined pharmacotherapy groups was 84.83% and 267.97% (IMI+β-HgS group), and 629.71% and 343.33% (SER+β-HgS group) higher than those of their corresponding single antidepressant-treated groups (IMI or SER group) in CA1 and DG regions (*p* < 0.01 or *p* < 0.001).

Next, the effects of stress and drug treatment on oligodendrocytes in the hippocampus were assessed. We measured the levels of NG2 (a marker for oligodendrocyte progenitors) and Olig2 (a pan-oligodendrocyte marker), respectively Compared with those in the control group, there was a dramatic loss of NG2+ and Olig2+ cells in the CA1, CA3, and DG regions of the hippocampus of mice in the model group (Figure 4D), and the fluorescence intensities of NG2+ and Olig2+ cells were both remarkably decreased (Figures 5B,C). Compared with those in the model group, NG2+ and Olig2+ cells had a larger distribution range in the hippocampal CA1, CA3, and DG regions in all drug-treatment groups. The fluorescence intensities of NG2+ cells were significantly higher than those in the control group in the hippocampal CA1, CA3, and DG regions in all drug-treatment groups, except the CA1 region in the SER+β-HgS group. The fluorescence intensities of Olig2+ cells were significantly higher than those of the control group in all three hippocampal subregions in both the β-HgS and IMI groups. Notably, the fluorescence intensity of NG2+ cells in the DG region of the IMI+β-HgS group was 142.56% higher than that in the IMI group (*p* < 0.001).

Neuronal proliferation in key hippocampal subregions was evaluated using a cell-cycle marker proliferating cell nuclear antigen (PCNA) and neuronal nuclear antigen neuron marker (NeuN). As shown in Figure 4D, the mice in the model group showed significantly weaker responses to PCNA and NeuN immunoreactivity in the hippocampal CA1, CA3, and DG regions compared with those in the control group. Compared with those in the model group, the fluorescence intensities of NeuN+ cells were significantly increased in all three hippocampal subregions in all drug-treatment groups (*p* < 0.05, *p* < 0.01, or *p* < 0.001). For the combined pharmacotherapy groups (IMI+β-HgS and SER+β-HgS groups), the fluorescence intensities of PCNA+ cells were significantly increased in all three hippocampal subregions (*p* < 0.01 or *p* < 0.001) compared with those in the model group. Furthermore, the fluorescence intensities of NeuN+ cells in the CA3 and DG regions, as well as PCNA+ cells in all three hippocampal subregions, in the SER+β-HgS group were significantly higher than those of the SER group (*p* < 0.05 or *p* < 0.001). Additionally, the fluorescence intensities of PCNA+ cells in all three hippocampal subregions of the IMI+β-HgS group were significantly higher than those of the IMI group (*p* < 0.001).

### 3.6 Effects on the contents of monoamine neurotransmitters, BDNF, and neuroendocrine hormone content in the hypothalamic-pituitary-adrenal axis

**FIGURE 6:**
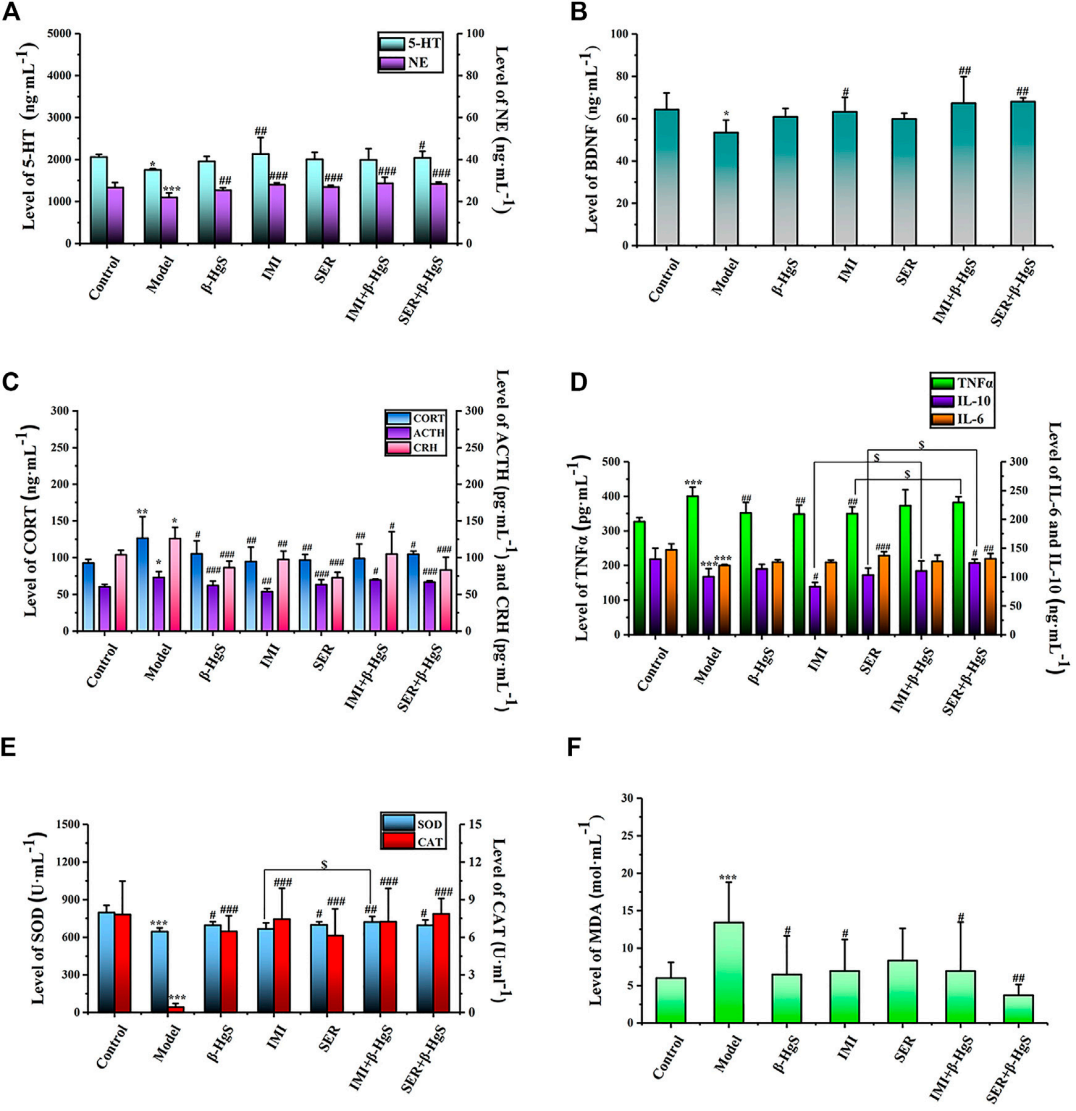
The effects of drug treatments on the levels of monoamine neurotransmitters **(A)** and BDNF **(B)**, and the biomarkers of HPA axis **(C)**, and the biomarkers of immune factors **(D)**, and **(E, F)** oxidative stress in stress-stimulated mice. Data are shown as the mean ± *SD* (*n* = 10). *p < 0.05, **p < 0.01, and ***p < 0.001 denote significant differences from the control group. ^#^ *p* < 0.05, ^##^ *p* < 0.01, and ^###^ *p* < 0.001 denote significant differences from the model group. ^$^ *p* < 0.05 denotes a significant difference from the relative monotherapy group.

Cortical levels of monoamine neurotransmitters (5-HT and NE), hippocampal levels of BDNF, and levels of three core neuroendocrine hormones (CRH, ACTH, and CORT) in the hypothalamic-pituitary-adrenal (HPA) axis were determined (Figures 6A–C). After three-week stress stimulation, cortical levels of 5-HT/NE and hippocampal levels of BDNF in model mice were significantly lower—whereas levels of CRH, ACTH, and CORT in the HPA axis were remarkably higher—than those in normal control mice (*p* < 0.05 or *p* < 0.01). Compared with those in the model group, the cortical levels NE, as well as the CRH, ACTH, and CORT levels in the HPA axis, were significantly increased in all drug-treated groups (*p* < 0.01 or *p* < 0.001). However, 5-HT levels were only significantly increased in the IMI and SER+β-HgS groups (*p* < 0.05 or *p* < 0.01), whereas BDNF levels were only significantly increased in the IMI, IMI+β-HgS, and SER+β-HgS groups (*p* < 0.05 or *p* < 0.01).

Among all groups, cortical 5-HT levels were the highest in the IMI group, cortical NE levels were the highest in the IMI+β-HgS group, and hippocampal BDNF levels were the highest in the SER+β-HgS group. Additionally, among all drug-treated groups, SER and IMI were the most effective at inhibiting the increases in levels of three core neuroendocrine hormones (CRH, ACTH, and CORT) induced by stress. Compared with those in the model group, CRH, ACTH, and CORT levels in the SER and IMI groups were decreased by 42.25%, 13.40%, and 23.63%, and by 22.67%, 26.31%, and 25.18%, respectively.

### 3.7 Effects on the contents of TNF-α, IL-6, and IL-10 content in serum

The effects of stress and drug treatments on immune inflammatory responses in mice were preliminarily investigated according to serum levels of TNF-α, IL-6, and IL-10. As shown in Figure 6D, after three-week stress stimulation, TNF-α levels in the model group were significantly higher—while IL-6 and IL-10 levels were both significantly lower—compared to those in the control group (*p* < 0.001). Compared with those in the model group, TNF-α levels were significantly decreased in the β-HgS, IMI, and SER groups (*p* < 0.01); IL-10 levels were significantly increased in the SER+β-HgS group (*p* < 0.05) but decreased in the IMI group (*p* < 0.05); IL-6 levels were significantly increased in the SER and SER+β-HgS groups (*p* < 0.01 or *p* < 0.001). Moreover, IL-10 levels in the IMI+β-HgS and SER+β-HgS groups were significantly higher than those in their corresponding IMI and SER groups (*p* < 0.05), whereas TNF-α levels in the SER+β-HgS group were significantly higher than those in the SER group (*p* < 0.05).

### 3.8 Effect on oxidative stress

To evaluate the oxidative stress levels of mice induced by three-week stress and drug treatments, serum levels of MDA, SOD, and CAT were determined (Figures 6E,F). Compared with those of the control group, MDA levels were significantly higher—whereas SOD and CAT levels were significantly lower—in the model group (*p* < 0.05). Compared with those of the model group, the MDA levels were significantly decreased after 3 weeks of drug treatment (except for the SER group), and CAT and SOD levels were significantly increased (except for the SOD content in the IMI group). Moreover, the MDA levels of the SER+β-HgS group were significantly lower than those of the SER group (*p* < 0.05), while SOD levels of the IMI+ β-HgS group were much higher than those of the IMI group (*p* < 0.05).

### 3.9 Effect on gut microbiota

#### 3.9.1 Diversity of gut microbiota

**FIGURE 7:**
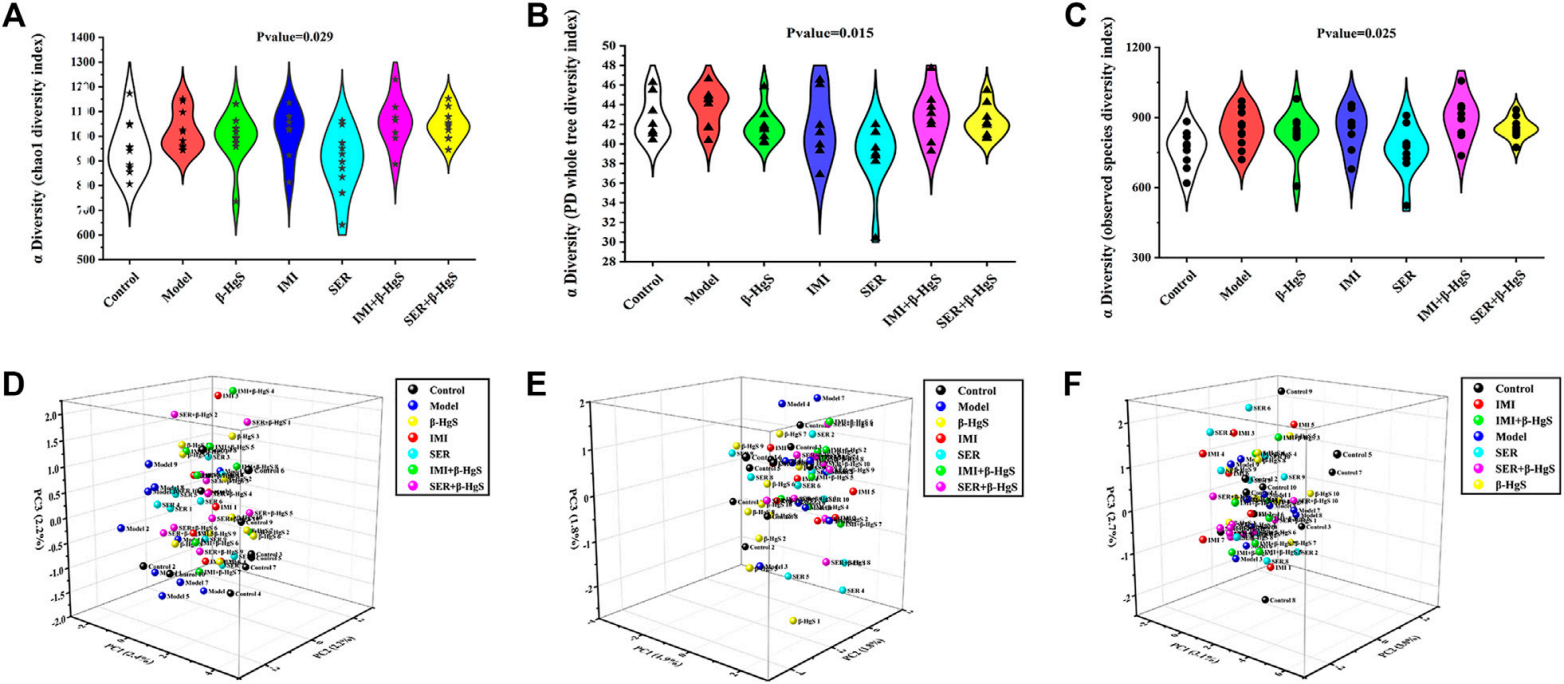
The diversity of gut microbiota. **(A)** Chao1 index, **(B)** PD whole tree index, **(C)** observed species, **(D)** Bray curtis distance, **(E)** Weighted Unifrac distance, **(F)** Unweighted Unifrac distance.

In the Alpha diversity analysis (Figures 7A–C), the differences between Chao1, PD whole tree index, and observed species were statistically significant (*p* < 0.05; *p* <0.05; *p* < 0.05). The analysis of beta diversity was calculated from the unweighted and weighted UniFrac distances, as well as the Bray–Curtis dissimilarity (Figures 7D–F; and Table 1). The results revealed that the bacterial microbiota of stress-treated mice were clustered apart from that of normal mice (*p*  < 0.05, multi-response permutation procedure analysis), and the diversity of gut microbiota also differed significantly among the drug-treatment groups (*A* > 0 and *p* < 0.05), indicating the varying degrees of significant effects on the diversity of gut microbiota induced by the five drug treatments.

**TABLE 1:** PCoA of gut microbiota beta diversity.

Beta diversity	*A* Value	Observed delta	Expected delta	*p* Value
Weighted unifrac distance	0.019097	0.263322	0.268449	0.064
Unweighted_unifrac distance	0.042404	0.335139	0.34998	0.001
Bray-Curtis dissimilarity	0.043234	0.537801	0.562103	0.001

#### 3.9.2 Altered composition in gut microbiota at the phylum level

**FIGURE 8:**
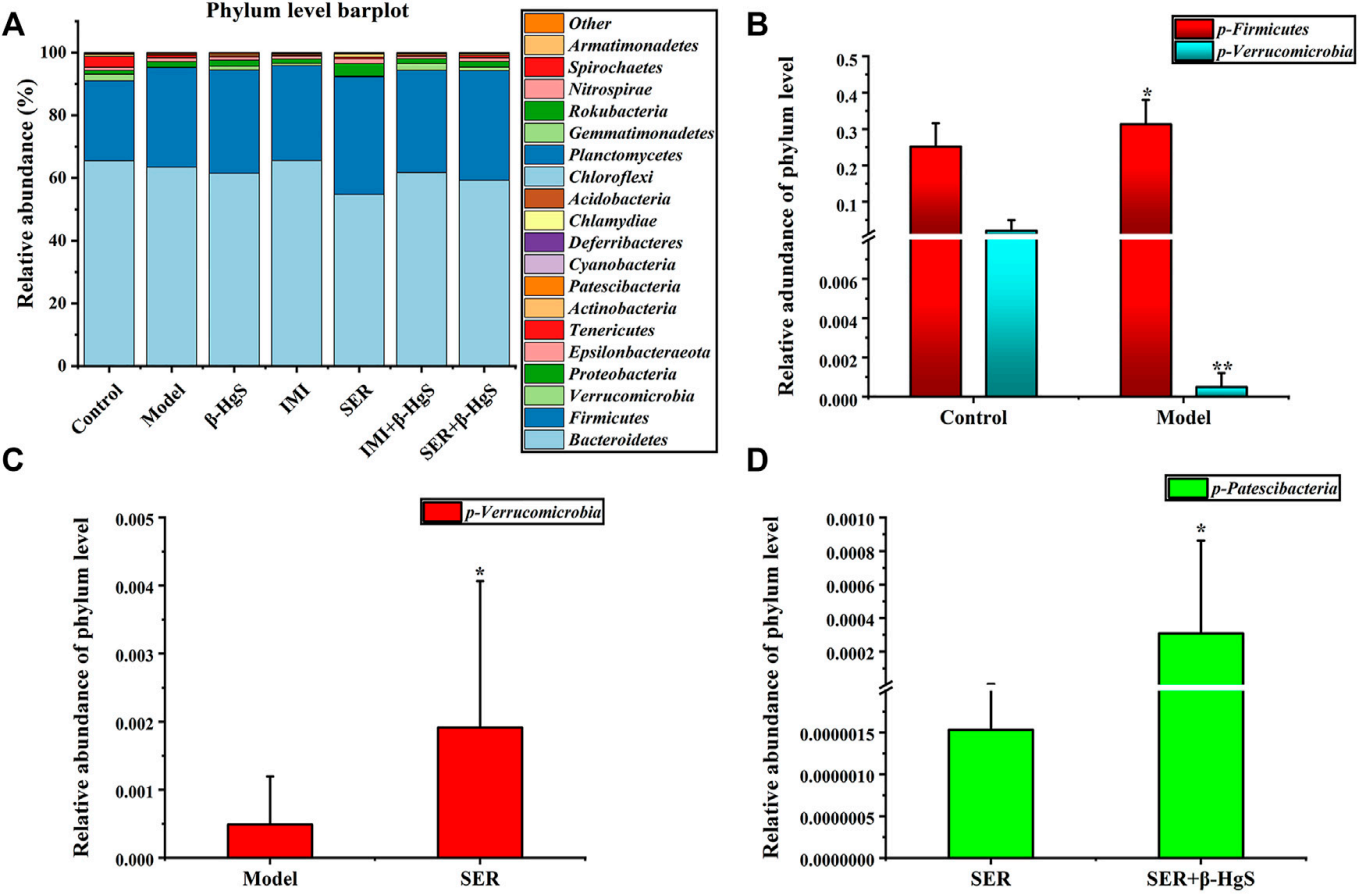
The effects of drug treatments on the composition of gut microbiota at the phylum level in stress-stimulated mice. **(A)** The relative abundances of phylum in intestinal contents of mice after 3 weeks of treatment. **(B)** Relative abundances of the gut microbiota with significant differences at the phylum level in the model group, compared with those in the control group. **(C)** Relative abundances of the gut microbiota with significant differences at the phylum level in the drug-treated groups, compared with those in the model group. **(D)** Relative abundances of gut microbiota with significant differences at the phylum level in the combination therapy group, compared with those of the relative monotherapy group. Data are shown as the mean ± *SD* (*n* = 10). *p < 0.05 and **p < 0.01 denote significant differences from the compared group.

Next, the phylum-level compositions of gut microbiota were identified (Figure 8A), and the differences in gut-microbiota abundances among the groups were analyzed by *t* tests (Figures 8B–E). The results showed that stress induced a significant increase in the relative abundance of *Firmicutes* and a significant decrease in the relative abundance of *Verrucomicrobia* in the model group (*p* < 0.05). Compared to that of the model group, the relative abundance of *Verrucomicrobia* was significantly increased in the SER and IMI+β-HgS groups (*p* < 0.05). Furthermore, the relative abundance of *Patescibacteria* in the SER+β-HgS group was significantly higher than that in the SER group (*p* < 0.05). However, the β-HgS, IMI, and SER+β-HgS groups showed no differences compared to the model group in terms of the phylum level of gut microbiota.

#### 3.9.3 Altered composition in gut microbiota at the order level

**FIGURE 9:**
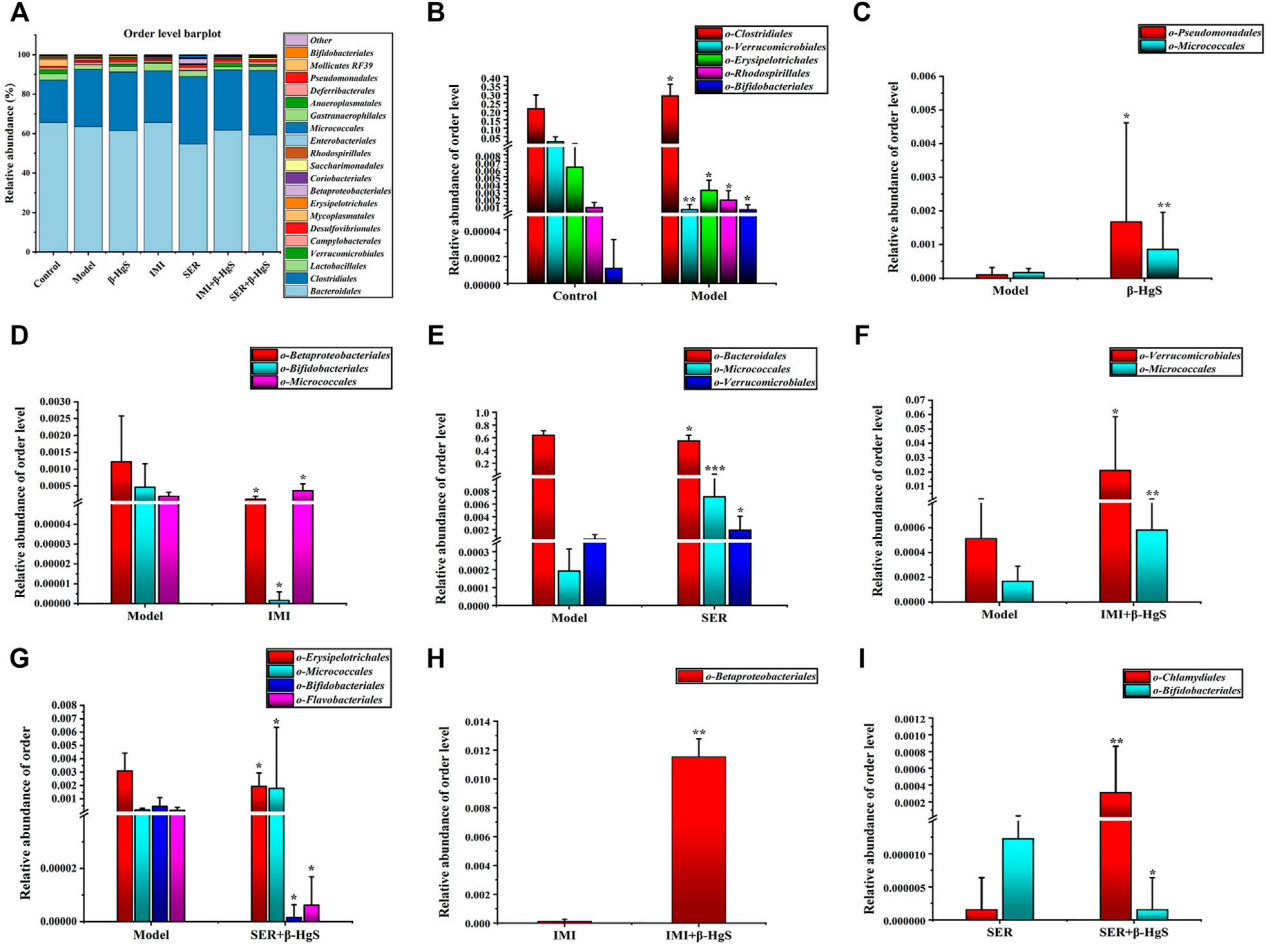
The effects of drug treatments on the composition of gut microbiota at the order level in stress-stimulated mice. **(A)** The relative abundances of constituents of gut microbiota at the order level in the intestinal contents of mice after 3 weeks of treatment. **(B)** Relative abundances of the gut microbiota with significant differences at the order level in the model group, compared with those in the control group. **(C–G)** Relative abundances of the gut microbiota with significant differences at the order level in the drug-treated groups, compared with those in the model group. **(H–I)** Relative abundances of the gut microbiota with significant differences at the order level in the combination therapy group, compared with those in the relative monotherapy group. Data are shown as the mean ± *SD* (*n* = 10). *p < 0.05, **p < 0.01, and ***p < 0.001 denote significant differences from the same kind of gut microbiota in the compared group.

Next, the order levels of gut microbiota were investigated (Figure 9A). *Clostridiales*, *Rhodospirillales*, and *Bifidobacteriales* levels were significantly higher and *Verrucomicrobiales* and *Erysipelotrichales* levels were significantly lower in the model group than those in the control group (*p* < 0.05) (Figure 9B). Compared with those in the model group, the relative abundances of two orders (*Pseudomonadales* and *Micrococcales*), one order (*Micrococcales*), two orders (*Micrococcales* and *Verrucomicrobiales*), two orders (*Micrococcales* and *Verrucomicrobiales*) and one order (*Micrococcales*) of bacteria were significantly higher (*p* < 0.05 or *p* < 0.01) in the β-HgS, IMI, SER, IMI+β-HgS, and SER+β-HgS groups, respectively (Figures 9D–G), whereas the relative abundances of two orders (*Betaproteobacteriales* and *Bifidobacteriales*), one order (*Bacteroidales*), and three orders (*Erysipelotrichales*, *Bifidobacteriales*, and *Flavobacteriales*) of bacteria were significantly lower (*p* < 0.05) in the IMI, SER, and SER+β-HgS groups, respectively. Notably, the relative abundances of *Betaproteobacteriales* in the IMI+β-HgS group and *Chlamydiales* in the SER+β-HgS group were significantly higher while that of *Bifidobacteriales* in the SER+β-HgS group was significantly lower—compared to those in the corresponding single antidepressant-treated groups (IMI or SER group) (*p* < 0.05).

#### 3.9.4 Altered composition of gut microbiota at the family level

**FIGURE 10:**
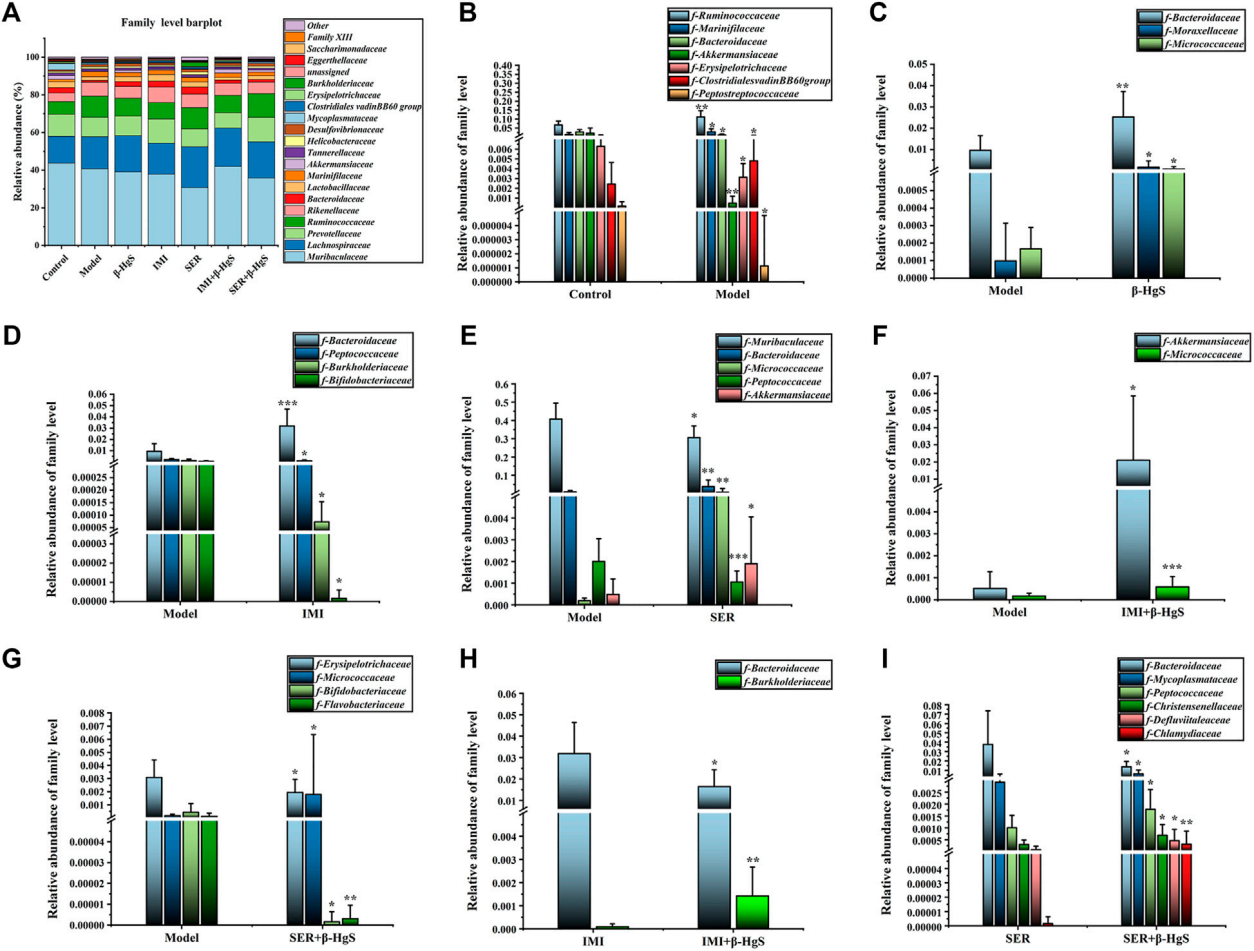
The effects of drug treatments on the composition of gut microbiota at the family level in stress-stimulated mice. **(A)** The relative abundances of constituents of gut microbiota at the family level in the intestinal contents of mice after 3 weeks of treatment. **(B)** The relative abundances of the gut microbiota with significant differences at the family level in the model group, compared with those in the control group. **(C–G)** The relative abundances of the gut microbiota with significant differences at the family level in the drug-treated groups, compared with those in the model group. **(H–I)** The relative abundances of gut microbiota with significant differences at the family level in the combination therapy group, compared with those in the relative monotherapy group. Data are shown as the mean ± *SD* (*n* = 10). *p < 0.05, **p < 0.01, and ***p < 0.001 denote significant differences from the same kind of gut microbiota in the compared group.

Next, the family levels of gut microbiota were investigated (Figure 10A). The results showed that *Ruminococcaceae*, *Marinifilaceae*, and *Clostridialesvadin BB60* presented at significantly higher levels, while *Bacteroidaceae*, *Akkermansiaceae*, *Erysipelotrichaceae*, and *Peptostreptococcaceae* presented at significantly lower levels in the model group than those in the control group (*p* < 0.05) (Figure 10B). Compared with those in the model group, the relative abundances of three families (*Bacteroidaceae*, *Moraxellaceae*, and *Micrococcaceae*), one family (*Bacteroidaceae*), three families (*Bacteroidaceae*, *Micrococcaceae*, and *Akkermansiaceae*), two families (*Micrococcaceae* and *Akkermansiaceae*), and one family (*Micrococcaceae*) of bacteria were significantly higher (*p* < 0.05, *p* < 0.01, or *p* < 0.001) in the β-HgS, IMI, SER, IMI+β-HgS, and SER+β-HgS groups, respectively, whereas three families (*Peptococcaceae*, *Burkholderiaceae*, and *Bifidobacteriaceae*), two families (*Muribaculaceae* and *Peptococcaceae*), and three families (*Erysipelotrichaceae*, *Bifidobacteriaceae*, and *Flavobacteriaceae*) of bacteria were significantly lower (*p* < 0.05) in the IMI, SER, and SER+β-HgS groups, respectively (Figures 10D–G). Compared with those in the corresponding single antidepressant-treated groups (IMI or SER group), the relative abundances of *Burkholderiaceae* in the IMI+β-HgS group and *Mycoplasmataceae*, *Peptococcaceae*, *Christensenellaceae*, *Defluviitaleaceae*, and *Chlamydiaceae* in the SER+β-HgS group were significantly higher (*p* < 0.05 or *p* < 0.01), whereas *Bacteroidaceae* in the IMI+β-HgS and SER+β-HgS groups was significantly lower (*p* < 0.05).

#### 3.9.5 Altered composition of gut microbiota at the genus level

**FIGURE 11:**
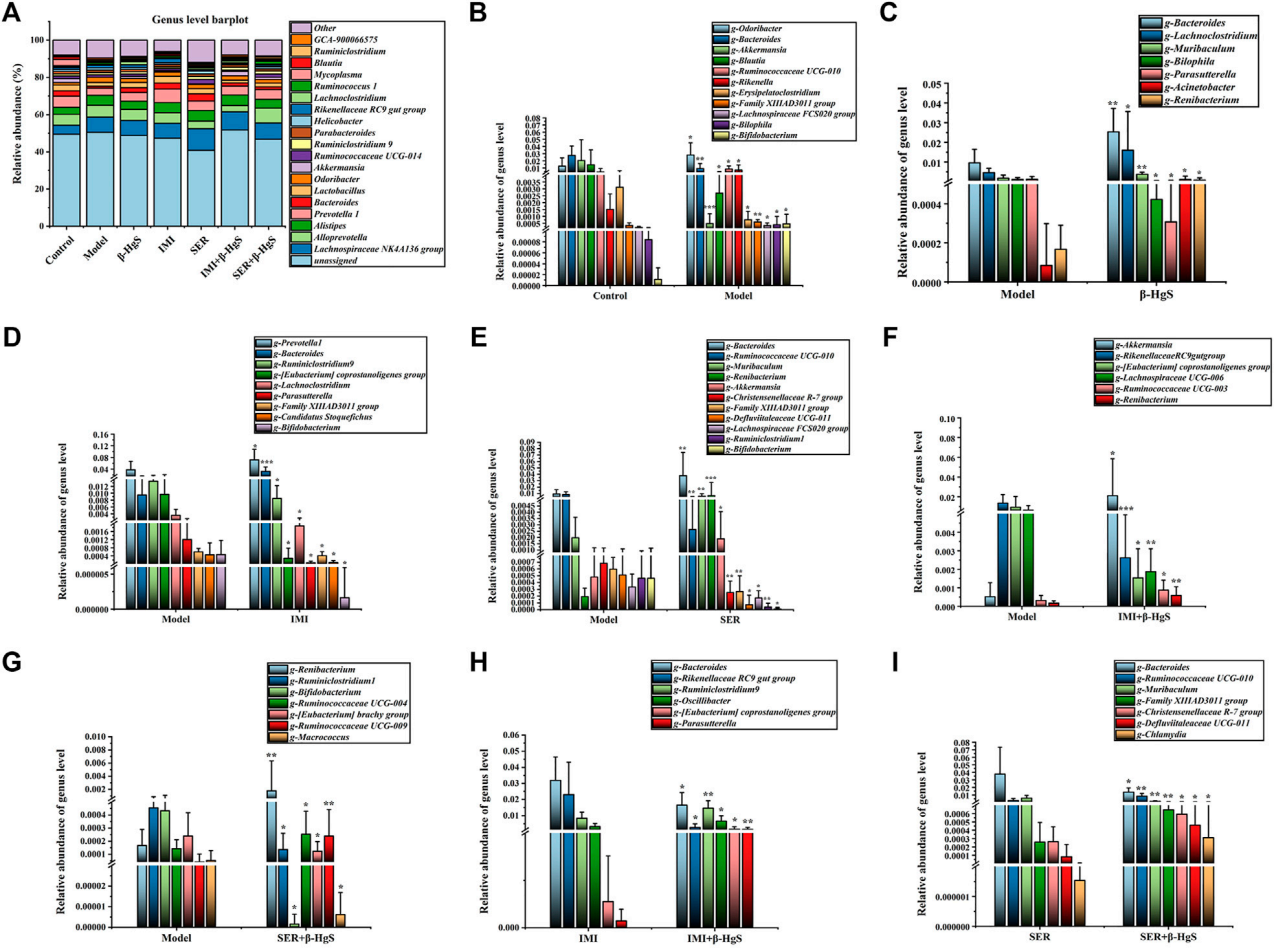
The effects of drug treatments on the composition of gut microbiota at the genus level in stress-stimulated mice. **(A)** The relative abundances of constituents of gut microbiota at the genus level in intestinal contents of mice after 3 weeks of treatment. **(B)** The relative abundances of the gut microbiota with significant differences at the genus level in the model group, compared with those of the control group. **(C–G)** The relative abundances of the gut microbiota with significant differences at the genus level in the drug-treated groups, compared with those of the model group. **(H–I)** The relative abundances of the gut microbiota with a significant difference at the genus level in the combination therapy group, compared with those of the relative monotherapy group. Data are shown as the mean ± *SD* (*n* = 10). *p < 0.05, **p < 0.01, and ***p < 0.001 denote significant differences from the same kind of gut microbiota in the compared group.

Next, the compositions of the gut microbiota at the genus level were investigated (Figure 11A). Compared with those of the control group, 11 genera of gut microbiota were significantly different in terms of their relative abundances in the model group (*p* < 0.05, *p* < 0.001, or *p* < 0.01). Specifically, the relative abundances of *Bacteroides*, *Akkermansia*, *Blautia*, and *Erysipelatoclostridium* were lower and those of *Odoribacter*, *Ruminococcaceae UCG-010*, *Rikenella*, *Family XIIIAD3011 group*, *Lachnospiraceae FCS020 group*, *Bilophila*, and *Bifidobacterium* were higher than those of the control group (Figure 11B). Compared with those of the model group, the relative abundances of five genera (*Bacteroides*, *Lachnoclostridium*, *Muribaculum*, *Acinetobacter*, and *Renibacterium*), two genera (*Prevotellal* and *Bacteroides*), four genera (*Bacteroides*, *Muribaculum*, *Renibacterium*, and *Akkermansia*), three genera (*Akkermansia*, *Ruminococcaceae UCC-009*, and *Renibacterium*), and three genera (*Renibacterium*, *Ruminococcaceael UCC-004*, and *Ruminococcaceae UCC-009*) were significantly higher (*p* < 0.05, *p* < 0.01 or *p* < 0.001), whereas those of two genera (*Bilophila* and *Parasutterella*), seven genera (*Ruminiclotridium*9, *[Eubacterium]coprostanoligenes group*, *Lachnoclostridium*, *Parasutterella*, *Family XIIIAD3011 group, Candidatus Stoquefichus*, and *Bifidobacterium*), seven genera (*Ruminococcaceae* UCG-010, *Christensenellaceae R-7 group*, *Family XIIIAD 3011 group*, *Defluviitaleaceae UCG-011*, *Lachnospiraceae FCS020 group*, *Ruminiclostridium1*, and *Bifidobacterium*), three genera (*Rikenellaceae RC9gut group*, *[Eubacterium] coprostanoligenes group*, and *Lachnospiracee UCG-006*), and four genera (*Ruminiclostridium*1, *Bifidobacterium*, *[Eubacterium] brachy group*, and *Macrococcus*) were significantly lower (*p* < 0.05, *p* < 0.01 or *p* < 0.001) in the β-HgS, IMI, SER, IMI+β-HgS, and SER+β-HgS groups, respectively (Figures 11C–G). Compared with those of the corresponding single antidepressant-treated groups (IMI or SER group), the relative abundances of *Ruminiclostridium9*, *[Eubacterium] coprostanoligenes group*, and *Parasutterella* in the IMI+β-HgS group as well as *Ruminococcaceae UCG-010*, *Family XIIIAD3011 group*, *Christensenellaceae R-7 group*, *Defluviitaleaceae UCG-011*, and *Chlamydia* in the SER+β-HgS group was significantly higher (*p* < 0.05 or *p* < 0.01), whereas those of *Bacteroides* and *Rikenellaceae RC9gut group* in IMI+β-HgS group and *Bacteroides* and *Muribaculum* in the SER+β-HgS group were significantly lower (*p* < 0.05) (Figures 11H,I).

#### 3.9.6 LDA Effect size analysis and functional prediction

**FIGURE 12:**
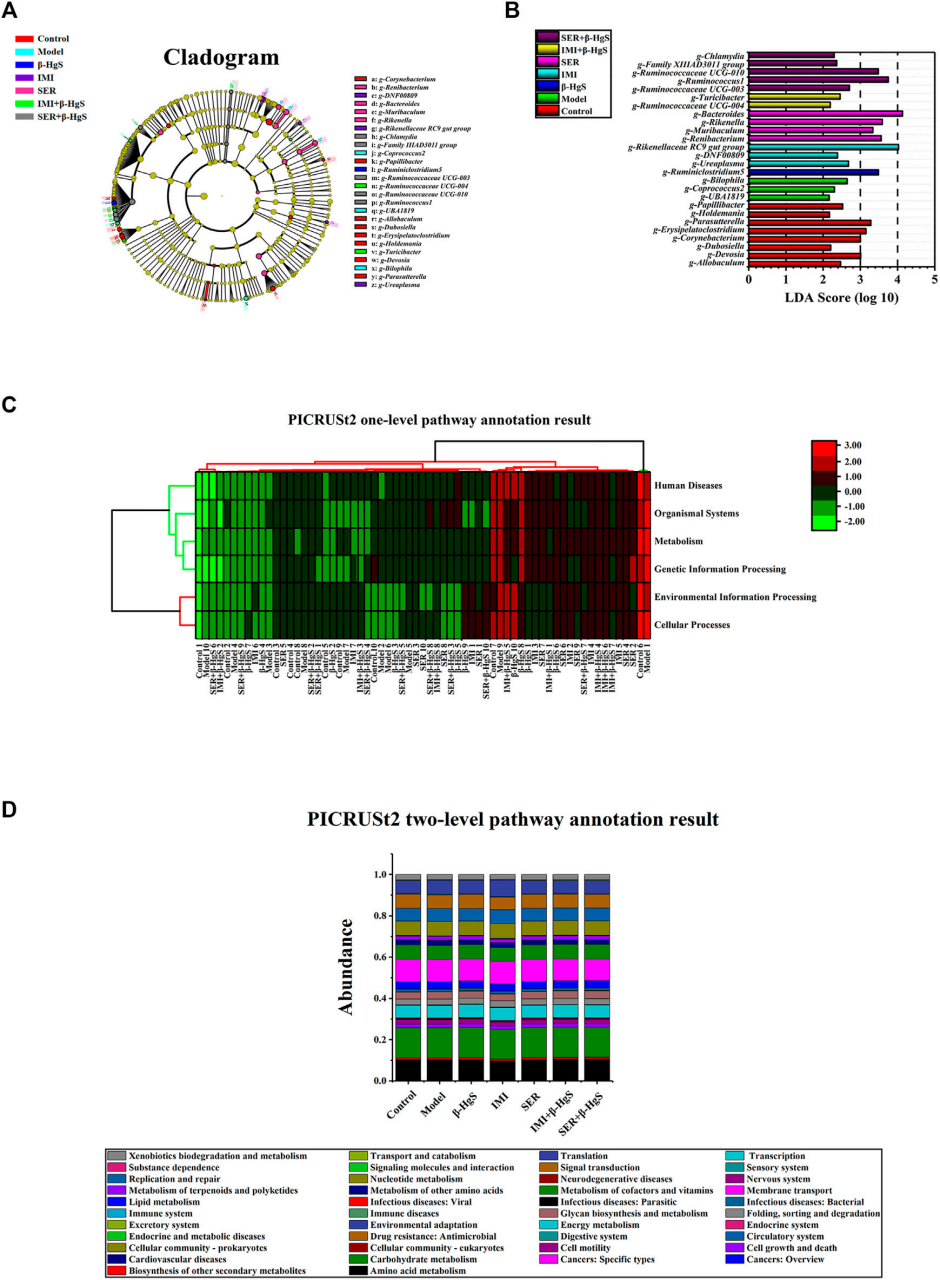
LEfSe analysis and functional prediction. **(A)** Cladogram representing taxa with different abundances of gut microbiota at baseline, and **(B)** histogram of linear discriminant analysis scores computed for differentially abundant features among groups, as well as **(C)** PICRUSt2 one-level pathway annotation results and **(D)** PICRUSt2 two-level pathway annotation results. The LDA score on the log10 scale is indicated at the bottom. The greater the LDA score, the more significant the phylotype biomarker is in the comparison.

As shown in Figure 12A, the microbial groups that play an important role in the grouping are separately displayed in the Cladogram. According to genus-level species statistical analysis, *Allobaculums*, *Dubosiella*, *Erysipelatoclostridium*, *Holdemania*, *Corynebacterium*, *Papillibacter*, *Devosia*, and *Parasutterella* are important gut microbiota for differentiation the control group; *Coprococcus2*, *UBA1819*, and *Bilophila* are important gut microbiota that differentiate the Model group; *Ruminiclostridium 5* was important gut microbiota that differentiated the β-HgS group; *DNF 00809*, *Rikenellaceae RC9 gut group*, and *Ureaplasma* are important gut microbiota that allow differentiation pressure in the IMI group; *Renibacterium*, *Bacteroides*, *Muribaculum*, and *Rikenella* are important gut microbiota that differentiate pressure in the SER group; *Ruminococcaceae* UCG-004 and *Turicibacter* are important gut microbiota that allow differentiation pressure in the IMI+β-HgS group; *Chlamydia*, *Family IIIAD3011 group*, *Ruminococcaceae UCG-003*, *Ruminococcaceae UCG-010* and *Ruminococcus1* are important gut microbiota that differentiate pressure in the SER+β-HgS group.

Moreover, according to the histogram of linear discriminant analysis (LDA) scores computed for differentially abundant features among the groups (Figure 12B), there were 8, 3, 1, 3, 4, 2, and 5 types of gut microbes with LDA values >3 in the Control, Model, β-HgS, IMI, SER, IMI+β-HgS, and SER+β-HgS groups, respectively. As shown in Figure 12C, the one-level pathways annotating gut microbiota are mainly distributed in Cellular Processes, Environmental Information Processing, Genetic Information Processing, Human Diseases, Metabolism, and Organismal Systems; the two-level pathway annotation (Figure 12D) distribution of the main pathways of gut flora Carbohydrate metabolism (14.4%–14.7%), Membrane transport (10.4%–10.9%), Amino acid metabolism (9.8%–10.6%), Translation (6.4%–7.9%), Nucleotide metabolism (7.0%–7.2%), Metabolism of cofactors and vitamins (6.6%–7.0%), Signal transduction (6.1%–6.7%) , Replication and repair (6.1%–6.6%) Energy metabolism (6.0%–6.2%).

### 3.10 Association analysis of differences between monotherapy and combination therapy

**FIGURE 13:**
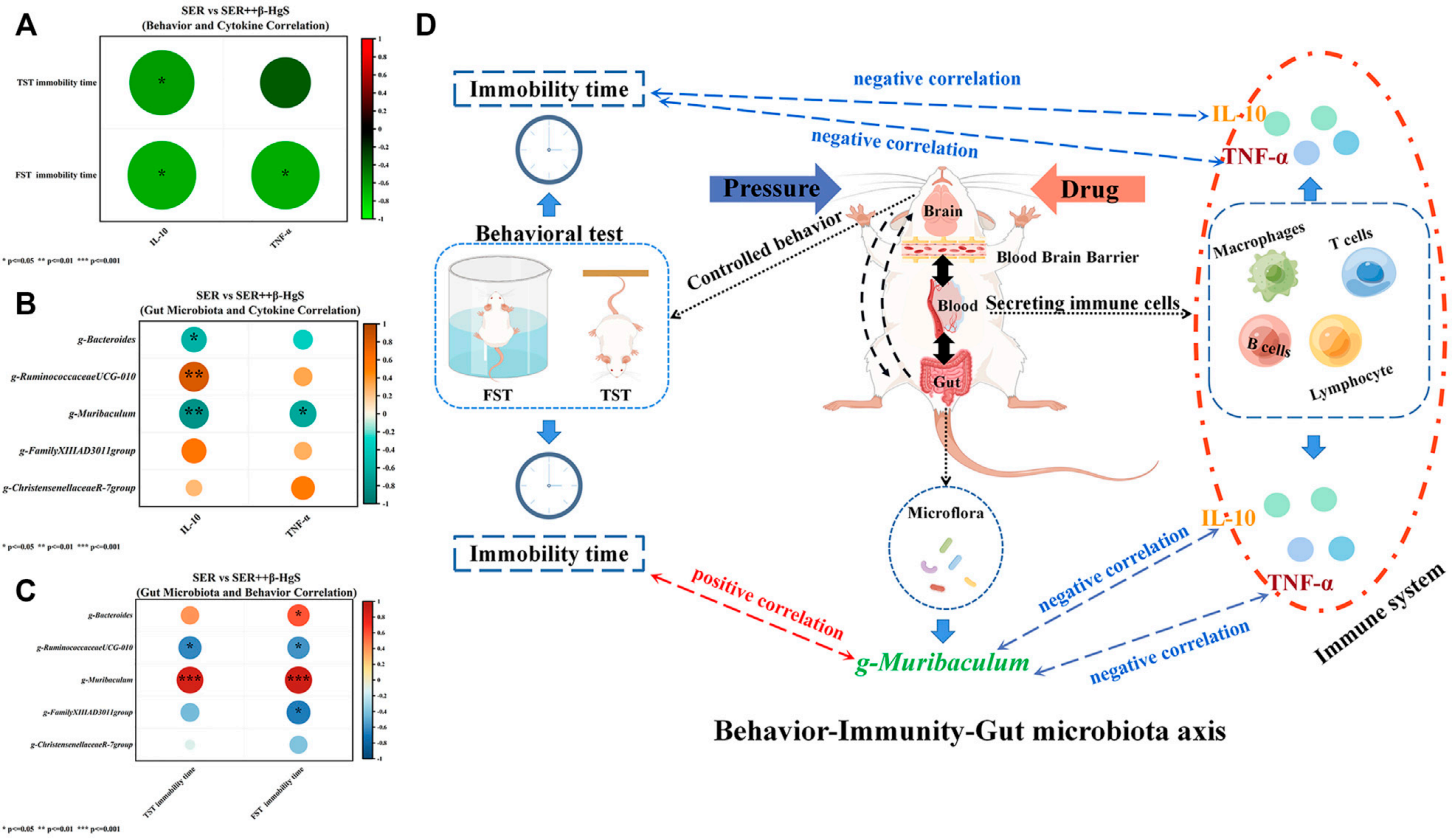
Pearson correlation analysis was performed on the differential physiological indicators, behavior and bacterial flora of SER vs. SER+β-HgS. **(A)** Behavior and Cytokine Correlation, **(B)** Gut Microbiota and Cytokine Correlation, **(C)** Gut Microbiota and Behavioral Correlation, **(D)** Behavior-Immunity-Gut microbiota axis.

Pearson correlation analysis was performed on the differential physiological indicators, behavior and bacterial flora of SER vs. SER+β-HgS, respectively, as shown in Figure 13. In behavior and cytokine correlation (Figure 13A), TST immobility time was negatively correlated with IL-10 (*p < 0.05*); FST immobility time was negatively correlated with IL-10 and TNF-α levels (*p < 0.05; p < 0.05*). IL-10 was negatively correlated with *Muribaculum* and *Bacteroides* (*p < 0.01*; *p < 0.05*) in differential microbiota and cytokine correlation (Figure 13B), and negatively correlated with *Ruminococccaceae UCG-010* (*p* < 0.01). In differential microbiota and behavioral correlation (Figure 13C), TST immobility time was positively correlated with *Muribaculum* (*p* < 0.001) and negatively correlated with *Ruminococccaceae UCG-010* (*p* < 0.01); FST immobility time was positively correlated with *Muribaculum*, *Bacteroides* (*p* < 0.001; *p* < 0.0*5*), and negatively correlated with *Ruminococccaceae UCG-010* and *Family XIIIAD3011 group* (*p <* 0.05; *p* < 0.05).

## 4 Discussion

According to existing studies, depression is mainly caused by mental stress, physical diseases and genetic factors, and most of the cases are caused by persistent mental stress (Marsay et al., 2018). In daily life, mental stress consists of two kinds of different stressors, predictable persistent stress and unpredictable short-term stress. Therefore, we used a combination of predictable and unpredictable stressors to simulate the depression-like environment of the mice used in this study. The results showed that the three-week combined action of predictable and unpredictable stressors induced depression-like symptoms in mice according to decreases in body weight, SPR, and 2 h-food consumption, as well as increases in immobility times in the FST and TST. Furthermore, these stressors inhibited exploratory behavior based on significant increases in RT and decreases in MT, CMT, and CMD in the OFT, all of which are typical depression-like behaviors (Harvey and Bouwer, 2000; Chourbaji et al., 2005; Peng et al., 2007; Rizvi et al., 2016). Additionally, mice in all drug-treatment groups had significantly higher body weights and SPRs (except for the body weights in the SER group), much more 2 h-food consumption, and less immobility times in the FST and TST compared with those of the model group, indicating less pleasure deficiency and learned helplessness compared to those in the model group. In particular, the combined therapy of SER and β-HgS showed more of an advantage than the single therapy of SER in preventing the learned helplessness induced by stress stimulation, according to significantly fewer immobility times in the FST and TST in the SER+β-HgS group. Additionally, mice in the SER+β-HgS group were the most active among all drug-treatment groups since only the SER+β-HgS group exhibited significantly less RT and more MT, CMT, and CMD in the OFT compared with these parameters in the model group. The above findings suggest that the depression-like symptoms of mice in the SER+β-HgS group were most relieved among those of all drug-treatment groups.

Over the past decade, a series of compelling studies have reported that long-term major depressive disorder is associated with central nervous system atrophy in the hippocampus. The hippocampus plays a crucial role in learning and memory, and changes in hippocampal volume are associated with cognitive deficits in major depression (Bremner et al., 2000; Sahay and Hen, 2007; Chen et al., 2015). These studies were careful and well-controlled, demonstrating hippocampal atrophy after controlling for total brain volume, independent of variables such as history of antidepressant treatment, electroconvulsive therapy, or alcohol consumption. In addition, patients with persistent depression have more severe atrophic hippocampus (Robert, 2001). In this study, after stimulation *via* predictable and unpredictable stressors for 3 weeks, mice in the model group exhibited significant atrophy in the hippocampus and structural damage in key hippocampal subregions. Moreover, compared with those in the control group, the GR levels and the number of oligodendrocytes (NG2 and Olig2 cells) were remarkably decreased, and neuronal proliferation (PCNA+ and NeuN+ expression) was significantly inhibited via stress stimulation in the model group. Compared with parameters in the model group, all drug-treatment groups showed less atrophy and structural damage in key hippocampal subregions. Compared with the effects of the corresponding single-antidepressant treatments, the combined therapy groups better promoted GR and neuronal proliferation levels in most key hippocampal subregions. It is worth noting that only the fluorescence intensity of NG2+ oligodendrocytes in the DG area of the IMI+β-HgS group was 142.56% higher than that of the IMI group (*p* < 0.001), but did not significantly increase the number of oligodendrocytes in other areas. Oligodendrocyte precursor cells (OPCs, also known as NG2-glia) are located in the adult gray and white matter parenchyma and constitute approximately 6% of the total number of cells in the central nervous system. Inflammation is a hallmark feature of many neurological diseases, and existing studies have found that OPCs have the ability to sense and respond to inflammation (Boccazzi et al., 2022). Under physiological conditions, OPCs play a crucial role in maintaining microglia homeostasis, and it has been demonstrated that depletion of OPCs can cause a decrease in the stability of microglia (Zhang et al., 2019; Liu and Aguzzi, 2020). Furthermore, NG2-glia ablation induced hippocampal neuronal cell death while increasing microglial activation and mRNA levels of interleukin-1β, interleukin-6, and tumor necrosis factor (TNF), pro-inflammatory factors that exacerbate inflammation , affecting the decline of neuroprotective hepatocyte growth factor (HGF) levels (Nakano et al., 2017). Therefore, it is believed that β-HgS may enhance the recovery of inflammation in the brain hippocampus of depressed mice by IMI by increasing NG2+ oligodendrocytes in the DG area.

Currently, several theories have been proposed about the mechanism of depression. The most common is monoamine transmitter deficiency, especially 5-HT and NE (Liu et al., 2017). Second, the HPA axis is over-excited, leading to dysfunction of the body (Thomas et al., 2019). Neurogenesis and neurodegeneration hypotheses are also important theories to explain the pathogenesis of depression (Sapolsky, 2004; Czéh and Lucassen, 2007). Finally, inflammatory response, a feature of many psychiatric diseases, also has a clear correlation with depression (Loftis et al., 2010). Furthermore, BDNF and GRs are generally considered to underlie the pathophysiology of MDD. BDNF plays a major role in neuronal growth and survival and is considered a neuromodulator (Popova and Naumenko, 2019). BDNF is also involved in neural plasticity, whereas decreases in GRs can result in HPA-axis dysfunction and hyperactivity. Finally, both BDNF and GFs show that neuroendocrine function is unusual in depressed individuals (Pariante and Lightman, 2008). Recent studies have found that oxidative stress plays an important role in the pathophysiological process of major depression through the action of free radicals, non-free radicals, reactive oxygen species, and reactive nitrogen; hence, oxidative stress products are important parameters for the measurement and prediction of depressive states (Chen et al., 2018; Miller et al., 2018). In this study, our data showed that three-week stress treatments in mice induced defects in the biogenic amine system, dysfunction of HPA axis and peripheral immune system, reduced neurogenesis, and aggravation of oxidative stress. Compared with those of the model group, the above features were alleviated in mice in the drug-treatment groups. In the drug-treatment groups, mice in the SER+β-HgS group had the largest effects of significantly alleviated aspects compared with those in the model group. In particular, the TNF-α and IL-10 content in the SER+β-HgS group was significantly lower than that in the SER group, indicating the superiority of the combined therapy of SER and β-HgS in increasing inflammation levels in mice. IL-10 is a potent anti-inflammatory cytokine released by immune and glial cells that drives the regulation of multiple anti-inflammatory processes (Roque et al., 2009; Kwilasz et al., 2015), and increasing evidence that pro-inflammatory cytokines are associated with psychiatric disorders, namely depression. Notably, recent studies have shown that anti-inflammatory cytokines, such as IL-10, can also modulate depressive-like behaviors, specifically, mice lacking IL-10 expression were shown to remain immobile for longer in FST (Mesquita et al., 2008). These results suggest that mice lacking IL-10 display more helplessness. Our behavioral test also showed this phenomenon, β-HgS can promote SRE to increase the level of IL-10, and IL-10 is negatively correlated with desperate behavior. Meanwhile, the direct effect of IL-10 in the central nervous system may be mediated by its role in regulating cell survival. IL10 prevents glial cell death (Molina‐Holgado et al., 2001; Strle et al., 2002) and increases the survival of cerebellar neurons (Bachis et al., 2001), however, we also found that β-HgS enhanced effects on IMI and SER restore neuronal proliferation in the hippocampus, and this phenomenon may also be related to the elevated level of IL-10, which needs further verification. Additionally, decreased IL-10 promotes an imbalance in the cytokine milieu that further activates the HPA axis, which induces the release of glucocorticoids to trigger pro-inflammatory effects (Sorrells and Sapolsky, 2007). Interestingly, we found that TNF-α was increased and IL-6 and IL-10 were decreased in stressed mice compared with unstressed mice. The results of this experiment showed that pressure stimulation caused an inflammatory response. Although IL-6 is a pro-inflammatory factor, the biological activity of IL-6 is not limited to this. In the current study, it was found that activated microglia produced IL-6, a pleiotropic cytokine, which plays an important role in the cytokine network. It can regulate the growth and differentiation of various cells, regulate immune response, acute phase response, and hematopoietic function, and play an important role in the body’s anti-infection immune response. As a neurotrophic factor related to neuroimmunity and neuroendocrine, IL-6 can stimulate neuronal process growth and neuron regeneration. Meanwhile, many studies have reported that IL-6 increases the prevalence of schizophrenia, and that IL-6 levels in serum of schizophrenia patients are higher than those of healthy controls. Elevated IL-6 induces enhanced activity of DA and 5-HT in hippocampus and prefrontal cortex. IL-6 was significantly elevated and highly correlated with major depressive disorder. Several studies noted that veterans with post-traumatic stress disorder (PTSD) had elevated levels of IL-6 in cerebrospinal fluid (CSF) but not in plasma compared to controls (Erta et al., 2012). Both IL-6 and TNF-α can increase the permeability of the blood-brain barrier, and blocking the effects of IL-6 and TNF-α can reduce stress-induced opening of the blood-brain barrier. On the contrary, the increase of IL-6 and TNF-α increases the permeability of the blood-brain barrier, which may help to accelerate the excretion of harmful substances in the brain and strengthen the two-way communication at the brain-blood level (Beurel et al., 2020). Therefore, we speculate that the decrease of IL-6 and TNF-α, under the dual stress stimulation of CRS + CUMS, is mostly manifested as a weakened neuroprotective effect, and at the same time, it reduces the stress-induced opening of the blood-brain barrier. After drug treatment, the levels of IL-6 and TNF-α increased, the permeability of the blood-brain barrier was increased, the two-way communication at the brain-blood level was enhanced, and the harmful substances in the brain were excreted faster. At the same time, IL-6 promotes neuron proliferation and protects nerves. Our experimental results showed that the levels of 5-HT and BDNF were increased in the combined treatment, which also supported our speculation. In addition, because the relationship between cellular immune factors and depression is not clear, in the drug treatment, the experimental results show that pro-inflammatory factors and anti-inflammatory factors are on the rise, but the stress group is one high and one low. We speculate that on the one hand, the levels of brain inflammation, serum inflammation and intestinal inflammation may not match in space and time, resulting in abnormal expression of pro-inflammatory and anti-inflammatory factors in serum. On the other hand, it may be related to the side effects of drugs Reaction-related, long-term use of antidepressant drugs, there is also the possibility of inducing inflammatory reactions.

Recently, the gut microbiome has been revealed to be crucial to human health *via* the bidirectional communication between the gut and the brain (known as the visceral-brain axis). Furthermore, many recent studies have demonstrated that some intestinal florae are associated with major depression (Evrensel and Ceylan, 2015); hence, the diversity of intestinal flora is considered to be helpful in understanding depression (Pusceddu et al., 2015). In this study, the relative abundances of gut microbes in mice in the model group exhibited significant changes in two, five, seven, and eleven categories at the phylum, order, family, and genus levels, respectively, compared with those of the control group. Among the gut microbiota with decreased relative abundances in the model group, *Bacteroides* are symbiotic bacteria in the intestine that produce short-chain fatty acids (SCFAs), such as butyrates, which can aid in maintaining normal intestinal permeability while suppressing mucosal inflammation. *Akkermansia* promotes the barrier function of the gut and the effectiveness of cancer immunotherapy. *Blautia* can help clear the intestinal tract of gas and may be associated with irritable bowel syndrome. *Erysipelatoclostridium* can be effective for treating and preventing diseases and conditions mediated by the IL-17 or Th17 pathways (Levine et al., 2018; Ansaldo et al., 2019; Schirmer et al., 2019). Among the gut microbiota with increased relative abundances in the model group, *Odoribacter* can produce acetic acid, propionic acid, and butyric acid in the gut. *Rikenellaceae* has been increased in septic rats, and the increasing amount of *Bilophila* may increase the risk of inflammatory bowel disease (IBD) and hepatobiliary disease (Hyland and Stanton, 2016; Li et al., 2017; Zheng et al., 2019).

Compared with those of the model group, the diversities of intestinal flora of mice in the drug-treatment groups were significantly altered, especially in the SER, IMI+β-HgS, and SER+β-HgS groups. Moreover, the diversities of intestinal flora of mice in the combined therapy groups were also different from those in the corresponding single antidepressant-treated groups. Compared with those in the corresponding single antidepressant-treated groups, mice in the IMI+β-HgS group had more *Ruminiclostridium* and *Ruminococcu* (two anaerobic bacteria that metabolize plant cell-wall polysaccharides and may play a positive role in treating Th17-driven inflammation) and *Parasutterella* (a member of the core gut microbiota that may play a role in microbial interactions and infection resistance, especially early in life) and less *Rikenellaceae* bacteria; mice in the SER+β-HgS group had more *Christensenellaceae* and *Defluviitaleaceae* (two probiotics that may negatively correlate with metabolic diseases such as fat deposition, IBD, and metabolic syndrome) and less *Muribaculaceae* bacteria (an anaerobic bacteria that produces succinate, acetate, and propionate, and its family is composed of metabolic guilds, each specializing in the degradation of particular types of polysaccharides) (Halfvarson et al., 2017; Ma et al., 2019; Smith et al., 2019). These changes in the gut microbiota may therefore represent one of the mechanisms by which β-HgS enhances the therapeutic effects of antidepressants in mice. Moreover, the relative abundance of *Bacteroides* in mice in the combined therapy groups was significantly lower than that in the corresponding single antidepressant-treated groups, indicating that β-HgS had an inhibiting effect on *Bacteroides*. We performed association analysis on the differences in physiological indicators, behavioral indicators and differential flora compared with the monotherapy and combination treatment groups. In SER vs. SER+β-HgS, gut microbiota *Muribaculum* was negatively correlated with despair behavior (TST and FST immobility time), and positively correlated with IL-10 and TNF-α. Meanwhile, IL-10 and TNF-α were negatively and positively associated with desperate behavior. This may indicate that β-HgS may enhance and improve the immune level and restore despair behavior abilities of depressed mice by affecting the abundance of the intestinal flora *Muribaculum*. Interestingly, in the time-honored Traditional Chinese Medicine (TCM), there are many ancient recipes that intentionally added mercury-containing ingredients for therapeutic purposes. Cinnabar (mercury sulfide, >96% HgS) is a representative mercury-containing mineral material used in traditional Chinese medicine formulations. Cinnabar is a naturally occurring mineral element with calming properties. According to the Chinese Pharmacopoeia (Halfvarson et al., 2017), traditional Chinese medicine formulations contain cinnabar (Zhao H. et al., 2018). Wansheng Huafengdan (WSHFD) is a traditional Chinese medicine used to treat nervous system diseases (Zhang et al., 2012). Cinnabar (HgS) is contained in WSHFD, and which can significantly attenuate LPS-induced DA uptake capacity and decrease in the number of TH-positive neurons, inhibit microglial activation, and improve LPS-induced TNFα, iNOS, IL-1β, and COX-2. However, WSHFD removal or reduction of cinnabar and realgar failed to prevent LPS-induced neurotoxicity, thus cinnabar and realgar have protective effects against LPS-induced neurotoxicity. Simultaneously, Angong Niuhuang Pill (AGNHW) is also a representative traditional Chinese medicine formulation containing realgar and cinnabar for treating acute ischemic stroke (Tsoi et al., 2019). Studies have found that AGNHW can reduce infarct volume, maintain blood-brain barrier integrity, and improve cerebral ischemia-reperfusion injury. However, removal of realgar and/or cinnabar from AGNHW abolished the neuroprotective effects, suggesting that realgar and cinnabar are essential elements for neuroprotective effects of AGNHW and synergistically act with other herbs. However, due to the complexity of the composition of Zuotai and the theory of Tibetan medicine, we acknowledge the limitations of this study and only explored the effects of clinically equivalent doses of the main components of the drug against antidepressants. The relationship between β-HgS and depression-related gut microbiota, behavior, and immune system remains unknown and requires further research.

## 5 Conclusion

Our present results suggest that β-HgS may promote the antidepressant effects of SER on depression-like symptoms in mice, possibly *via* ameliorating learned helplessness, as well as atrophy and severe structural damage of the hippocampus, induced by stress stimulation. Our pathogenic studies showed that β-HgS enhanced the effects of SER on promoting GR and neuronal proliferation levels in key hippocampal subregions, as well as increased serum IL-10 and TNF-α levels in stress-stimulated mice. As for IMI, β-HgS enhanced its effects in preventing atrophy and severe structural damage in the hippocampus, promoting GR and neuronal proliferation levels in key hippocampal subregions and increasing serum IL-10 and SOD levels, whereas β-HgS did not significantly enhance the ameliorating effect of IMI on depression-like behavior in stress-stimulated mice. Furthermore, these combination therapies resulted in increased changes in the diversity of important gut microbiota compared to corresponding monotherapies, with associations between *Muribaculum* relative abundance and desperate behavior and cytokine levels (Figure 12D), which may be a factor in the enhancement of such combination therapies. Important factors in the efficacy of these combination therapies include SER or IMI for β-HgS.

## Data Availability

The raw data supporting the conclusions of this article will be made available by the authors, without undue reservation.
